# Atomic force microscopy as analytical tool to study physico-mechanical properties of intestinal cells

**DOI:** 10.3762/bjnano.6.151

**Published:** 2015-07-06

**Authors:** Christa Schimpel, Oliver Werzer, Eleonore Fröhlich, Gerd Leitinger, Markus Absenger-Novak, Birgit Teubl, Andreas Zimmer, Eva Roblegg

**Affiliations:** 1 Institute of Pharmaceutical Sciences, Department of Pharmaceutical Technology, NAWI Graz, Karl-Franzens-University of Graz, BioTechMed-Graz, Austria; 2 Medical University of Graz, Center for Medical Research, BioTechMed-Graz, Austria; 3 Research Unit Electron Microscopic Techniques, Institute of Cell Biology, Histology and Embryology, Center for Medical Research, Medical University of Graz, BioTechMed-Graz, Austria; 4 Research Center Pharmaceutical Engineering GmbH, Graz, Austria

**Keywords:** atomic force microscopy, Caco-2 cells, elasticity, M cells, mechanical properties

## Abstract

The small intestine is a complex system that carries out various functions. The main function of enterocytes is absorption of nutrients, whereas membranous cells (M cells) are responsible for delivering antigens/foreign substances to the mucosal lymphoid tissues. However, to get a fundamental understanding of how cellular structures contribute to physiological processes, precise knowledge about surface morphologies, cytoskeleton organizations and biomechanical properties is necessary. Atomic force microscopy (AFM) was used here as a powerful tool to study surface topographies of Caco-2 cells and M cells. Furthermore, cell elasticity (i.e., the mechanical response of a cell on a tip indentation), was elucidated by force curve measurements. Besides elasticity, adhesion was evaluated by recording the attraction and repulsion forces between the tip and the cell surface. Organization of F-actin networks were investigated via phalloidin labeling and visualization was performed with confocal laser scanning fluorescence microscopy (CLSM) and scanning electron microscopy (SEM). The results of these various experimental techniques revealed significant differences in the cytoskeleton/microvilli arrangements and F-actin organization. Caco-2 cells displayed densely packed F-actin bundles covering the entire cell surface, indicating the formation of a well-differentiated brush border. In contrast, in M cells actins were arranged as short and/or truncated thin villi, only available at the cell edge. The elasticity of M cells was 1.7-fold higher compared to Caco-2 cells and increased significantly from the cell periphery to the nuclear region. Since elasticity can be directly linked to cell adhesion, M cells showed higher adhesion forces than Caco-2 cells. The combination of distinct experimental techniques shows that morphological differences between Caco-2 cells and M cells correlate with mechanical cell properties and provide useful information to understand physiological processes/mechanisms in the small intestine.

## Introduction

The human small intestine consists of a cell monolayer, which is predominantly composed of enterocytes mixed with mucus-secreting goblet cells [1]. Apart from enterocytes, membranous epithelial cells (M cells) reside throughout the small intestine as follicular-associated epithelium (FAE) that overlays lymphoid follicles (e.g., Peyer's patches) [2]. One of the most prominent features of epithelial enterocytes are the microvilli that cover the cell surface and form the so-called intestinal brush border [3]. The brush border membrane provides a greatly expanded absorptive surface, which facilitates rapid absorption of digestive products [4], but also constitutes an effective barrier against microorganisms, pathogens and foreign substances [5]. Moreover, assembly of the F-actin network in the brush border occurs due to expression and recruitment of actin-binding proteins [6]. The main proteins involved are fimbrin and villin, whereby the latter one is the key component and determines organization and plasticity of the F-actin network [7–8]. In contrast, M cells show no brush border with only sparse irregular microvilli [9–10]. Interestingly, in M cells villin accumulates in the cytoplasm and thus does neither induce extensive microvillus growth nor brush border formation [11]. The mechanism behind this is still unknown. It is suggested that villin either controls gelation of F-actin or that other proteins are involved [3,12], which block brush boarder assembly [13]. Thus, it is likely that variations in cell morphology between enterocytes and M cells may lead to differences in their physico-mechanical properties (elasticity, adhesion), which, as a consequence might impact certain cellular processes.

Apart from magnetic twisting cytometry (MTC) [14–15], micropipette aspiration [16] and magnetic/optical tweezers or optical traps [17–19], atomic force microcopy (AFM) is a versatile and potent tool for studying biological structures [20–22]. AFM enables both topographical and force curve measurements (atomic force spectroscopy) [23]. The former allow getting an image of the cell surface to observe its morphological and structural features. The latter is used to study elastic properties of a cell. Briefly, the central part of an AFM is a sharp tip, situated at the end of a flexible cantilever. The reflection of a laser beam focused at the back side of the cantilever is used to measure the movement of the tip. When the probe at the end of the cantilever interacts with the sample surface, the laser light pathway changes and is finally detected by a photodiode detector. The measured cantilever deflections vary (depending on the sample nature, i.e., high features on the sample cause the cantilever to deflect more) hence, a map of surface topography can be generated [21–22,24]. Moreover, quantitative analysis of the cell elasticity is possible by analyzing force-distance curves via monitoring the response of a cantilever once the tip is pushed against the plasma membranes. As a consequence, indentation occurs. The amount of force acting on the cantilever as a function of indentation enables an estimation of the nanomechanical properties of living cells, such as elasticity and adhesion [21,25–27].

To get a basic understanding regarding surface morphologies, mechanical properties and cytoskeleton organizations, enterocytes (Caco-2 cells) and M cells were studied in an in vitro co-culture model [28]. For this, enterocytes were cultured with Raji B cells to trigger M cell formation. AFM was used as a tool to study surface topography, elasticity and adhesion. Moreover, differences in F-actin networks were investigated via phalloidin labeling using confocal laser scanning fluorescence microscopy (CLSM) and scanning electron microscopy (SEM).

## Results and Discussion

### Morphological surface structures and cytoskeleton organization of Caco-2 cells and M cells

Cells display various surface architectures, which enable them to carry out different functions. For example, the FAE mainly consists of two cell types: absorptive enterocytes with a brush border and M cells without this highly organized apical specialization. The main function of enterocytes is the absorption of nutrients. M cells on the other hand provide an portal through which antigens/microorganisms can be delivered to the underlying mucosal lymphoid tissues [29]. This is due to the fact that M cells show a higher endocytic and transcytotic capacity than enterocytes. Hence, the fundamental question arises whether this is also reflected by the physico-mechanical properties of their respective cell surfaces.

**Figure 1:**
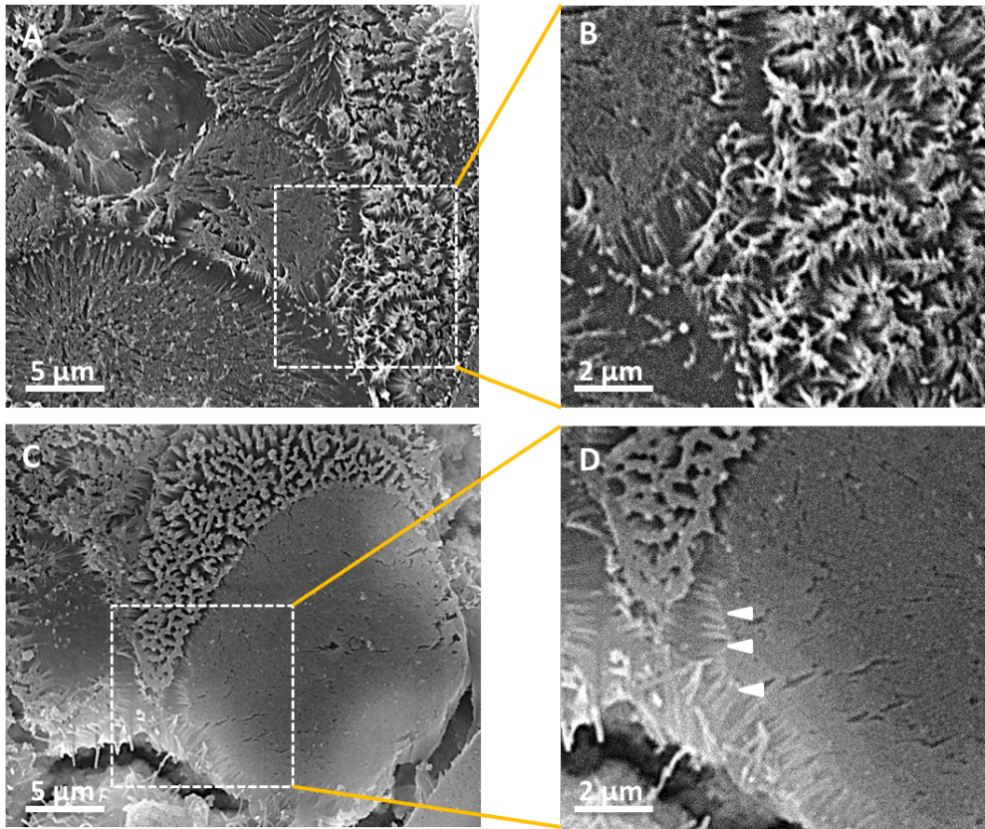
SEM analyses of the Caco-2 monolayer (A, B) and the Caco-2/M cell co-culture (C, D). The most prominent features on the Caco-2 cell surfaces are the microvilli that cover the surface forming the typical intestinal brush border (A, B). In contrast, M cells lack in microvilli (C). Arrowheads indicate sparse truncated microvillar structures on the edge of the cell membrane of a M cell (D).

SEM was used in order to firstly verify differentiation of Caco-2 cells to M cells in the presence of Raji B cells and secondly to evaluate differences in shape/surface morphology between both cell types. The SEM images show that the absorptive surface of Caco-2 cells is covered with densely packed microvilli, indicating the formation of a well-differentiated brush border structure (Figure 1A,B). In contrast, the apical surface of M cells is nearly devoid of microvilli. The few remaining villi that render the apical surface membrane appear to be sparse, short and/or truncated (Figure 1C,D). These findings are in excellent agreement with Owen et al., Bockman et al. and Gebert et al. [30–32]. They demonstrated that microvilli are less regular in M cells than in Caco-2 cells, differing in lengths as well as diameters. This suggests that the absence of a well-developed brush border in M cells may facilitate the adherence of antigens on the cell surface and, as a consequence, cellular uptake processes [2]. By contrast, the large surface area of intestinal microvilli is more appropriate for terminal digestion and absorption of soluble nutrients, electrolytes and water [33].

Although SEM typically provides nanometer resolution images of cell surfaces, a major drawback of this technique is that imaging usually requires fixation, drying and sputter coating of the samples. However, there are advanced SEM technologies available, such as environmental scanning electron microscopy (ESEM), which does not require complex sample preparation [34]. This allows preserving and analyzing of biological samples/structures at a hydrated state most closely approximating the native state. Although ESEM presents some additional beneficial features, considerable disadvantages including a high signal-to-noise ratio and/or limited resolution may arise [35].

**Figure 2:**
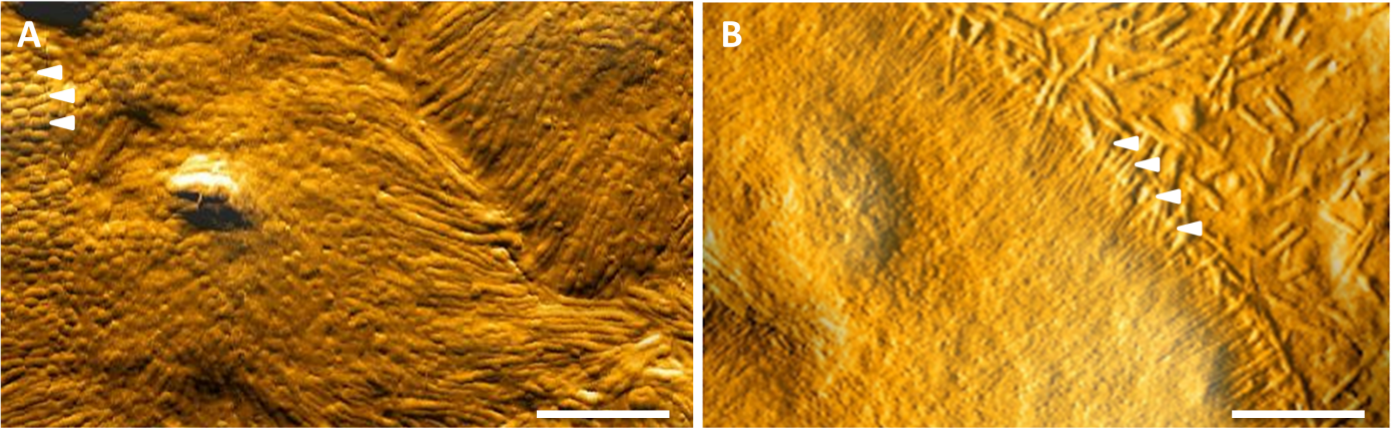
Topographic AFM images of a Caco-2 cell (A) and a M cell (B). The well-differentiated brush border of epithelial Caco-2 cells (A) depicts a densely packed array of upright orientated microvilli (marked by arrowheads) covering the entire surface. In contrast, the M cell surface (B) is supported by sparse truncated microvilli (marked by arrowheads) which appear shorter than those found in Caco-2 cells (scale bar = 5 µm).

In contrast, AFM allows high resolution (topographical) imaging of cells under (semi)hydrated, unfixed physiological conditions. Hence, complicated specimen preparation as well as destruction of native molecular conformations/structures can be avoided [36]. With this in mind, AFM was used in contact mode to explore the surface morphology of Caco-2 and M cells in more detailed. Unfortunately, it was not possible to localize M cells in the co-culture, since the large hydrated cells were highly flexible and only rough cell contours could be detected. Thus, cells were cultivated on transwell^®^ inserts and scanned in contact mode in a semi-dry state at ambient temperatures [37–38]. The results revealed that Caco-2 cells show the typical microvilli-rich intestinal brush border upon reaching confluence. Highly densely packed microvilli projecting perpendicular to the surface (marked by arrowheads in Figure 2A) were detected. In contrast, M cells depict a flat surface and comprise only short truncated microvilli that form an arch around the edge of the M cell (see Figure 2B). Moreover, the microvilli observed in M cells appear to be rudimentary and limp.

Each microvillus consists of a bundle of 20 F-actin microfilaments containing several actin-binding proteins, such as fimbrin and villin. Villin serves as F-actin cross-linker, and is thus responsible for polymerization of monomeric actin to microfilaments and/or the linkage of single microfilaments into hexagonally packed bundles [6,39]. According to the literature, villin is localized at the apex of cells that display a well-developed brush border [40]. In M cells, however, villin was found to be diffusely distributed in the cytoplasm and no microvillus growth and brush border assembly was induced [8,11,41–43]. This is in accordance with the absence of defined microvilli at the outer M-cell surfaces as verified by the SEM and AFM images.

**Figure 3:**
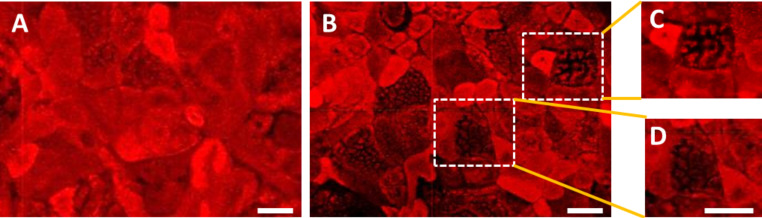
Optical images of the cytoskeleton organization in Caco-2 cells (A) and M cells (B–D). F-actin was stained with rhodamine-phalloidin. Caco-2 cells depict a well-differentiated brush border indicated by the intense red F-actin staining. In contrast, M cells show a reduced/absent brush border indicated by a reduced F-actin labeling (B–D) (scale bar = 20 µm).

To further verify that the different number of microvilli reflects an altered organization of the F-actin network between M cells and Caco-2 cells labeling of cytoskeletal F-actin-fibers with rhodamine-phalloidin was performed. In Caco-2 cells, an intense F-actin labeling at the apex of the cells was obtained, indicating a fully developed brush border (Figure 3A). In contrast, F-actin staining at the apex of M cells was markedly decreased due to a reduced or absent brush border (Figure 3B–D).

### Elasticity (force-indentation) measurements of Caco-2 cells and M cells

Villin is not only involved in the formation and/or regulation of the actin cytoskeleton, it also controls gelation of F-actin by inducing bundling of actin-filaments and thereby assures the stability of microvilli [42]. Hence, it is likely that differences in the mechanical properties of Caco-2 and M cells, such as elasticity and adhesion, might occur. To study this in detail, atomic force spectroscopy was used. For local force curve (indentation) measurements, the tip of the cantilever was placed over the location of interest (i.e., peripheral region/cell edge, nuclear area, cell body/cytoplasm) and the mechanical response was recorded as the cantilever was moved toward the cell surface. Such force–indentation curves of Caco-2 cells and M cells revealed variations of elastic values dependent on the cell location that was investigated. Generally, cells were more compliant at the nuclear area and became stiffer towards the periphery. Due to the higher compliance in the proximity of the cell nucleus, the loading force applied by the cantilever resulted in an increased elastic indentation of the cell by the tip due to an enlarged contact area. In contrast, indentation values obtained at positions at the cell edge were reduced.

**Figure 4:**
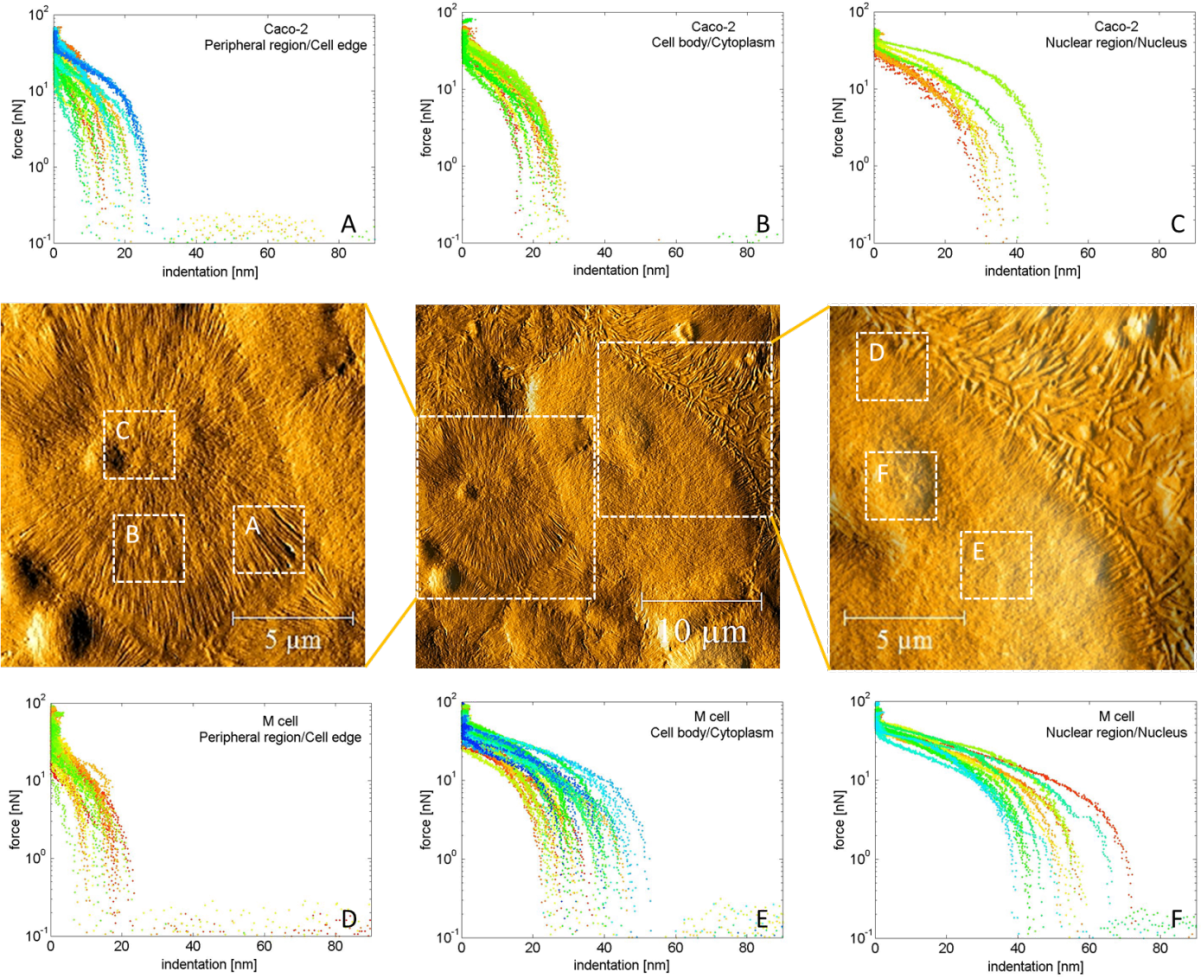
Force–indentation curves and topographical images of a Caco-2 cell (A–C) and a M cell (D–F) classified into peripheral region/cell edge, nuclear area and cell body/cytoplasm.

This is in accordance with previous studies [44–45]. It was shown in astrocytes (glial cells) that the elastic modulus near the nuclear region was an order of magnitude higher than at the edge of the cell. However, Caco-2 cells showed a 1.7-fold reduced elasticity compared to M cells (Figure 4A–F). Specifically at regions near the nucleus, M cells revealed a significantly higher elasticity than Caco-2 cells (see Figure 4). These increased elasticity values in M cells can be attributed to a decrease in filamentous actin. During the descent of a cell from a Caco-2 cell to a M cell, the cytoskeletal structure changes, more precisely F-actin-rich microvilli forming the intestinal brush border disappear, leading to an increased compliance compared to Caco-2 cells. This is also consistent with previous results, where actin was found to be reduced by 30% in cancerous keratinocytes compared to normal keratinocytes, which consequently leads to a decreased compliance of cancer cells [46].

Moreover, macrophages, which are also immune cells, display a similar arrangement of F-actin-rich structures, also referred as podosomes [47]. In activated state, podosomes rearrange and form a belt-shaped structure (i.e., rosette) on the outer surface of the cellular membrane. The rosette triggers migration and phagocytic processes and shows a 5-fold decreased elasticity compared to podosome-free regions (nuclear area). This is in accordance with our study. Since M cells are also immune cells, it seems that the arrangement of the sparse truncated microvilli and the increased elasticity at nuclear M cell regions (3-fold compared to peripheral region) are likely to be responsible for their high endocytic and transcytotic capacity. Reduced elasticity values at the cell periphery of Caco-2 cells can be explained by cell-cell junctions. Caco-2 cells form a dense monolayer with transepithelial electric resistance values (TEER) of 422 ± 8.77 Ω·cm^2^. This is due to the fact that cells are connected via tight junctions (TJs), which are very strong junctions that lack in intercellular spaces (compared to gap junctions or desmosomes) due to fusion of the outerleaflets of the membranes of adjacent cells. They are responsible to maintain the integrity of the cell layer, which is likely to be associated with the cell mechanics such as high cell stiffness and reduced cell elasticity at the cell periphery. In contrast, once enterocytes are interdispersed with M cells TEER values (388 ± 2.74 Ω·cm^2^) decrease. Studies reported by Clark and Hirst [48] and Gebert et al. [2] showed that TJs in non-FAE intestinal epithelia differ from FAE TJs. M cells show an increased depth and an altered arrangement of TJ strands. Thus, we speculate that this is reflected in the cell mechanics, such as a higher elasticity values compared to Caco-2 cells. Moreover, it is reported that the density of epithelial cell monolayers impacts cell mechanics (as well as the dynamics) due to variations of compressive forces [49–50].

**Figure 5:**
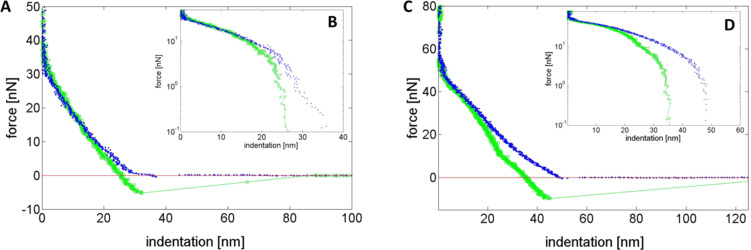
Representative force–indentation curves of a Caco-2 cell (A, B) and a M cell (C, D). The inset shows the force–deformation curve of the same indentation data on a logarithmic scale.

To deeper understand the obtained elastic properties in the nuclear regions, representative sample force curves of Caco-2 cells and M cells were selected from the force map presented in Figure 4. One way to analyze force–indentation curves in more detail is to investigate the difference between the approaching and retracting part, which are parameters that reflect the plastic and/or elastic (deformation) behavior of the sample under load. For a mechanical response, which is ideally elastic, the indentation and retraction curve will be identical (overlap). Cells undergoing plastic deformations (i.e., the cell membrane is non-reversible distorted during increasing load) result in significantly changed retraction forces [21]. The results of our study showed that force-indentation/retraction curves nearly overlapped in both cell types, indicating a mechanical response, which is dominated by elastic contributions at large indentation. This confirms that the cell integrity remains on the contact with the sharp cantilever (Figure 5). At very low indentations, both cell types show plastic deformation but this effect is more significant in M cells (see Figure 5C,D). This can be explained by higher indentation values obtained for M cells (50 nm) compared to Caco-2 cells (30 nm). Basically, the Hertz model has been validated for indentation analysis of cell mechanical properties, providing a single parameter called the Young´s modulus of the cell. However, this model assumes that the tested sample is homogenous, linear elastic, isotropic and continuously thick [51–52]. Related to eukaryotic cells, none of these requirements apply. The microvillar structures are responsible for a rough and heterogonous cell surface. The cytoskeleton comprises accessory proteins (e.g., villin) that induce formation of F-actin filament bundles and control the length of F-actin filaments [53], resulting in non-linear elasticity. Thus, we alternatively displayed the indentation values in the nanometer range taking into account distinct cell locations of Caco-2 cells and M cells.

### Evaluation of the attraction/repulsion (adhesion) forces

Elastic properties of cells can be directly linked to cell adhesion, since indentation also determines the number of adhesive bonds which are formed between cells and a surface. Hence, a smaller indentation and a consequent reduced contact area leads to a decrease in cellular adhesion [54]. Thus, we zoomed into the region, where the cantilever got in contact with the sample. Due to strong adhesion forces (van der Waals forces), the tip snapped in contact with the cell membrane. When retracting the tip, adhesion was maintained until the cantilever-force overcame the pull-off force (also referred as adhesion force) [51]. Lowest adhesion forces were found at the periphery of Caco-2 cells and slightly increased in the nuclear regions. However, in M cells adhesion was significantly higher, particularly in the nuclear region. This can be explained by the surface morphology and by the cell elasticity. M cells exhibit a smooth and more elastic surface due to the absence of microvilli, resulting in a significantly higher adhesion ability compared to rough Caco-2 cells [55]. Apart from surface nature, adhesive interactions of cells with other surfaces available in the (intestinal) environment are usually mediated by adhesion complexes/receptors. Such adhesion receptors include members of the cadherin, immunglobulin, selectin, proteoglycan and integrin superfamilies [56]. They trigger signaling pathways, which are involved in cellular processes (e.g., cell survival, tissue organization, binding interactions, specificity of cell–cell interactions) [56–58]. However, the cell adhesion molecule α5β1 integrin exhibits a different distribution pattern in M cells compared to enterocytes. Enterocytes display integrin only on basolateral and lateral surfaces, whereas M cells express α5β1 integrin on the apical membrane [41]. It is known that this cell adhesion molecule assists not only transformation from enterocytes to M cells but also preferential uptake by M cells [59]. This suggests that the presence of α5β1 integrin on the apical surfaces of M cells is likely to be responsible for the enhanced adhesion properties obtained via AFM.

However, it has to be added that AFM measurements performed under semi-dried conditions also show limitations, since physiological conditions are not fully reflected but are likely to change the interface between the gut lumen and the brush boarder membrane. Intestinal mucus, for instance, is continuously secreted by goblet cells and forms an efficient acellular barrier that strongly impacts adhesive interactions between intestinal epithelial cells and diverse substances/antigens. Due to intake of food, differences in the pH occur, which leads to changes in the viscoelastic properties of the mucus layer. Apart from food residuals, higher concentrations of digestive enzymes are available in the human small intestinal environment that influence transport processes through the mucus layer into the underlying tissue. Notably, bile salts, which are amphiphilic chemical derivates of cholesterol act as permeation enhancer via altering of the cell membrane integrity [60]. Moreover, bile salts form micells in aequeous solutions, enhancing transport of foreign substances [61].

This clearly shows that further research activities (e.g.; liquid-state AFM imaging using simulated intestinal fluid) are required to fill remaining data gaps on the effects of these parameters on cell mechanics/kinetics and, as a consequence, on cellular uptake processes (e.g., nanoparticulate systems/antigens).

## Conclusion

The current study shows that cytoskeletal structures and the content of F-actin filaments strongly impact nanomechanical properties (i.e., elasticity, adhesion) of intestinal cells. In Caco-2 cells, F-actin filaments are organized as densely packed bundles forming a well-differentiated brush border. In M cells, F-actin filaments are arranged as short and limp structures in the cell periphery resulting in microvilli that form an arch around the edge of M cells. These morphological differences correlate with the cell elasticity: Caco-2 cells show a 1.7-fold reduced elasticity compared to M cells. Moreover, elasticity of M cells increased significantly from the cell edge to the nuclear region. Since elastic properties of cells can be directly linked to cell adhesion, adhesion to the smooth and more elastic surface of M cells is enhanced, thus, facilitating the adherence of antigens and, as a consequence, cellular uptake processes.

## Experimental

### Cell cultures

Raji B cells were a kind gift from R. Fuchs (Medical University of Graz, Austria) and were grown in RPMI 1640 medium supplemented with 10% fetal bovine serum (FBS) (Invitrogen GmbH, Darmstadt, Germany), 1% non-essential amino acids (NEAA) (Invitrogen GmbH, Darmstadt, Germany), 1% L-glutamine (Invitrogen GmbH, Darmstadt, Germany) and 1% penicillin and streptomycin (PenStrep) (Invitrogen GmbH, Darmstadt, Germany) at 37 °C in a humified 5% CO_2_ atmosphere. Cells were cultured as previously described [62]. Caco-2 cells (ACC169, HTB-37 clone from the German Collection of Microorganisms and Cell Cultures) were cultivated at 37 °C under 10% CO_2_ water saturated atmosphere in complete medium consisting of Dulbecco´s Modified Eagle Medium (DMEM) supplemented with 10% FBS, 1% NEAA, 1% L-glutamine and 1% PenStrep according to the protocol of des Rieux et al. [1].

For experimental studies the double culture (Caco-2/Raji B co-culture), comprising enterocytes and M cells, was co-cultivated following previously described protocols [1,28]. Briefly, 5 × 10^5^ Caco-2 cells (passage 8–20) suspended in 0.5 mL supplemented DMEM were seeded onto polycarbonate 12-well Transwell^®^ filters (Corning Incorporated, USA; 3 µm mean pore size, 1.12 cm^2^ surface area). Caco-2 cells were maintained under standard incubation conditions for 14–16 days and medium both on the apical (0.5 mL) and basolateral side (1.7 mL) was changed every other day. Subsequently, 5 × 10^5^ Raji B cells (passage 8–20), resuspended in supplemented DMEM were added to the basolateral compartment of inserts promoting the differentiation of M cells. Cell monolayer integrity and confluence were evaluated by measuring the transepithelial electrical resistance (TEER) with an Endohm culture cup connected to an epithelial volt ohm meter (World Precisions Instruments, Sarasota, USA). For AFM cell imaging/force spectroscopy inserts were washed thrice with PBS, cut and mounted on round (15 mm) glass coverslips. This coverslip containing semi-dried cells was mounted in a Quick Change Imaging Chamber RC-40LP (Warner Instruments, USA) and measured directly afterwards.

### Scanning electron microscopy

In addition, scanning electron microscopy (SEM) was used to evaluate morphological changes of cell surface architectures and examine protrusive surface structures including microvilli. For this, specimens were prepared similar as described previously [62]. After cultivation in transwell^®^ systems cells were washed twice with PBS. Fixation was performed in Schaffer´s fixative (37% formol/100% ethanol) for 2 h [63]. Subsequently, fixed samples were dehydrated through a graded series of ethanol (80%, 90%, 100%), incubating for 20 min at room temperature in each ethanol grade. Subsequently, samples were dried with hexamethyldisilazane and the removed filter membranes were given a thin coating of gold palladium (Bal-Tec SCD 500) to improve the surface conductance of the sample and thus avoid surface charging of the sample under the beam. The samples were sputtered at 25 mA for 60 s under argon atmosphere and images were acquired using a scanning electron microscope (Zeiss DSM 950).

### Topographic imaging using atomic force microscopy (AFM)

The topography of different cell types (i.e., Caco-2 cells and M cells) was investigated using a Nanosurf AFM with an Easyscan2 controller (Switzerland). All measurements were performed using a ContAl-G cantilever (Budgetsensors, Romania) with an aluminum coating. Topography measurements were performed in contact mode at a line scan rate of 0.5 s/line. Various scan sizes revealed information on different length scales. As we were unable to localize M cells in the co-culture, due to highly hydrated and flexible cells, measurements were performed in a semi-dry state as demonstrated elsewhere [37–38].

### Tetramethylrhodamine (TRITC)-phalloidin staining

Visualization of the cytoskeletal F-actin network was performed using TRITC-phalloidin (Invitrogen GmbH, Darmstadt, Germany) in a similar manner as described earlier in literature [64]. In brief, cells were quickly rinsed in warm phosphate buffered saline (PBS; 0.01 M phosphate buffer, 0.15 M NaCl, pH 7.4) and fixed with 4% formaldehyde in PBS for 15 min at room temperature (RT). Next, cells were washed with PBS and permeabilized for 5 min at RT with 0.1% Triton X-100 in PBS. Subsequently, TRITC-phalloidin was added and cells were incubated for 20 min at RT in the dark. For CLSM imaging inserts were extensively washed and mounted on glass slides. Phalloidin-TRITC dyed cells were detected at 543 nm excitation wavelength using a LP 560 nm band pass detection for the red channel and images were examined with CLSM (Zeiss LSM 510 META) equipped with equipped with ZEN software (Zeiss Germany).

### Atomic force spectroscopy and indentation force measurements

The mechanical properties of the cells were obtained via force curve measurements, (i.e., the deflection of the cantilever as function of the indentation was detected). Similar to the imaging, measurements were performed in a semi-dry state as demonstrated elsewhere [37–38]. The experimental data were recalculated allowing the force acting on the cantilever (respectively the cell) to be determined. For all calculations a cantilever spring constant of 0.1 N/m was assumed (specified by the manufactures). A matlab program based on Butt et al. [65] was used for data handling and plotting.
